# Comparison of Two Strategies to Generate Antigen-Specific Human Monoclonal Antibodies: Which Method to Choose for Which Purpose?

**DOI:** 10.3389/fimmu.2021.660037

**Published:** 2021-05-04

**Authors:** Anna M. Ehlers, Constance F. den Hartog Jager, Tineke Kardol-Hoefnagel, Miriam M.D. Katsburg, André C. Knulst, Henny G. Otten

**Affiliations:** ^1^ Center for Translational Immunology, University Medical Center Utrecht, Utrecht University, Utrecht, Netherlands; ^2^ Department of Dermatology/Allergology, University Medical Center Utrecht, Utrecht University, Utrecht, Netherlands

**Keywords:** monoclonal antibodies, single cell sequencing, immortalization, limiting dilution, antigen-specific B cells

## Abstract

Human monoclonal antibodies (mAbs) are valuable tools to link genetic information with functional features and to provide a platform for conformational epitope mapping. Additionally, combined data on genetic and functional features provide a valuable mosaic for systems immunology approaches. Strategies to generate human mAbs from peripheral blood have been described and used in several studies including single cell sequencing of antigen-binding B cells and the establishment of antigen-specific monoclonal Epstein-Barr Virus (EBV) immortalized lymphoblastoid cell lines (LCLs). However, direct comparisons of these two strategies are scarce. Hence, we sought to set up these two strategies in our laboratory using peanut 2S albumins (allergens) and the autoantigen anti-Rho guanosine diphosphate dissociation inhibitor 2 (RhoGDI2, alternatively ‘ARHGDIB’) as antigen targets to directly compare these strategies regarding costs, time expenditure, recovery, throughput and complexity. Regarding single cell sequencing, up to 50% of corresponding V(D)J gene transcripts were successfully amplified of which 54% were successfully cloned into expression vectors used for heterologous expression. Seventy-five percent of heterologously expressed mAbs showed specific binding to peanut 2S albumins resulting in an overall recovery of 20.3%, which may be increased to around 29% by ordering gene sequences commercially for antibody cloning. In comparison, the establishment of monoclonal EBV-LCLs showed a lower overall recovery of around 17.6%. Heterologous expression of a mAb carrying the same variable region as its native counterpart showed comparable concentration-dependent binding abilities. By directly comparing those two strategies, single cell sequencing allows a broad examination of antigen-binding mAbs in a moderate-throughput manner, while the establishment of monoclonal EBV-LCLs is a powerful tool to select a small number of highly reactive mAbs restricted to certain B cell subpopulations. Overall, both strategies, initially set-up for peanut 2S albumins, are suitable to obtain human mAbs and they are easily transferrable to other target antigens as shown for ARHGDIB.

## Introduction

Antibody diversity enables the adaptive immune system to generate a humoral response against virtually any antigen. Gene recombination of variable (V), diversity (D) and joining (J) gene segments for the heavy chain and V and J gene segments for the corresponding light chain results in a wide variety of antibodies with distinct specificities. The diversity is even enlarged by imprecision during the V(D)J gene rearrangement process (1, 2). The introduction of somatic hypermutations is an additional tool to increase diversity but more importantly to strengthen antibody’s affinity against the respective target (3).

Disease-related specific antibody repertoires are often studied by comparing specific B cell subpopulations between patients and healthy donors using next generation sequencing (4–7). This powerful approach, however, does not provide any information about antigen reactivity, affinity and functionality. For this reason, several studies included the generation of human monoclonal antibodies (mAbs) in order to assess their functionality and to map their characteristics related to their genetic features (8, 9). Besides identifying genetic features associated with health or disease, human mAbs can support the mapping of conformational epitopes formed by closely located amino acids upon folding (9). While linear epitopes, comprised of sequential amino acids, can easily be mapped by e.g. peptide microarrays, the mapping of conformational epitopes requires more sophisticated techniques like mass spectrometry, nuclear magnetic resonance spectroscopy and/or mutation libraries (10). Since these techniques can hardly be executed with patient serum containing polyclonal antibodies, human mAbs are powerful tools to overcome this obstacle. Moreover, data obtained with mAbs derived from humans are thought to be more easily translatable to clinical research compared to data obtained with e.g. mouse-derived mAbs (9, 11). Additionally, these genetic and functional data can provide a valuable mosaic for systems immunology approaches, which model the complexity of the immune system *in silico*.

The first human mAbs were obtained in the early 90’s by phage display technology using single chain or Fab fragment libraries (12, 13). Nearly simultaneously, transgenic animals consisting of human immunoglobulin genes provided an additional tool (14). These approaches, however, are artificial and cannot represent a complete human antibody repertoire, emphasizing the advantage of mAbs generated from human peripheral blood or tissues. The first strategy to generate human mAbs from peripheral blood included the establishment of immortalized B cell lines by Epstein-Barr Virus (EBV) infection followed by limiting dilution cloning (15, 16). More recently, the immortalization by EBV was partly replaced by BCl-6 overexpression mimicking a germinal center status accompanied by constant antibody secretion (17). The most recent technique, however, is single cell sequencing with subsequent heterologous antibody expression (18).

**Figure 1:**
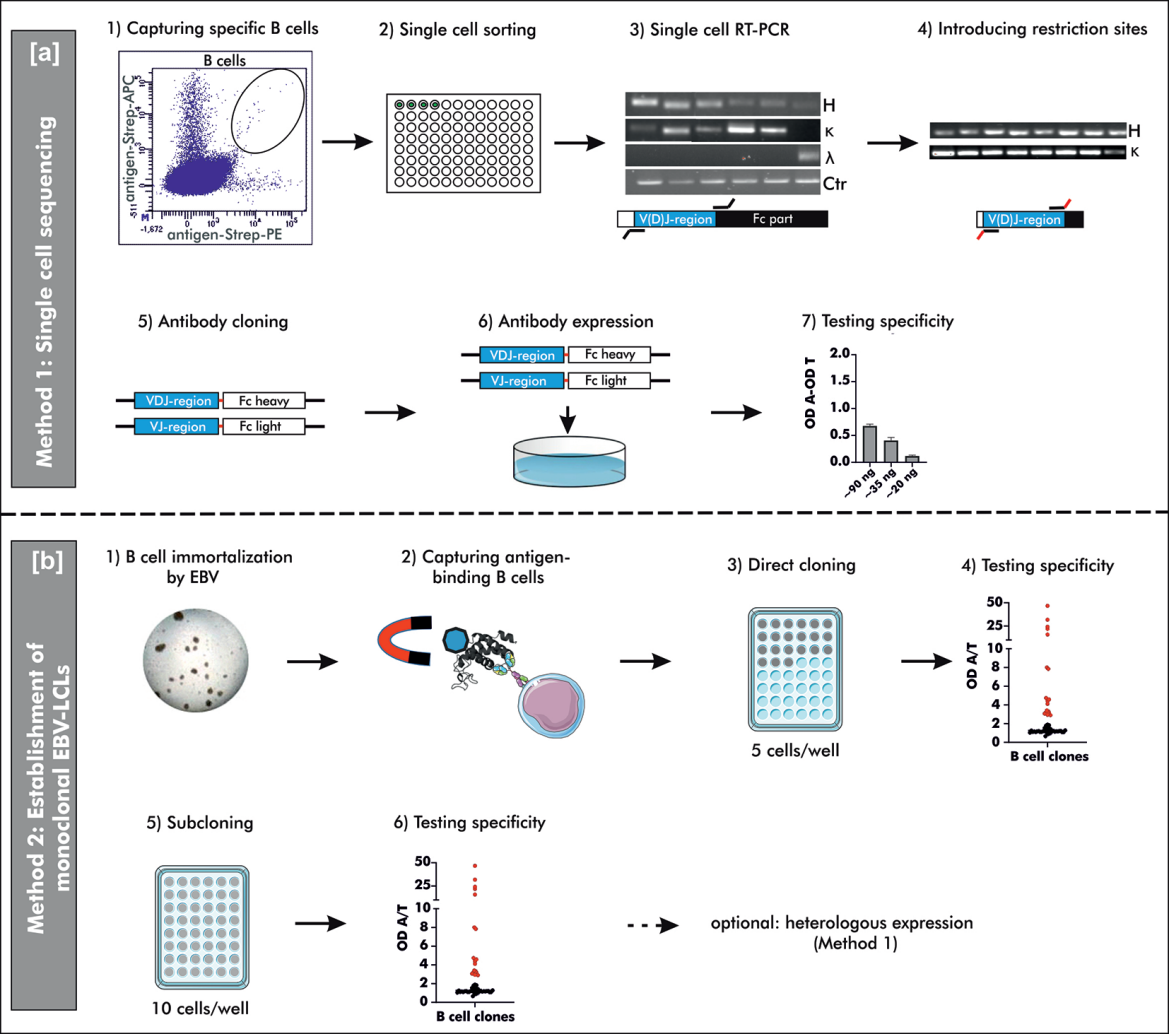
Schematic overview on two different methods to generate human mAbs. **(A)** Method 1: “Single cell sequencing”: B cells for single cell sorting were captured by double-positive antigen-tetramer staining (1 + 2). Upon transcription of total mRNA into cDNA, the V(D)J gene transcripts of the heavy and corresponding light chain were amplified by reverse transcriptase polymerase chain reaction (RT-PCR) using multiplex primers (3). The V(D)J gene usage was determined by Sanger sequencing and respective specific simplex primers were used to introduce restriction sites for subsequent cloning (4). Amplified V(D)J gene transcripts were cloned into commercially available pFUSEss-IgH vectors (Invivogen) carrying either the heavy or light chain constant backbone (Fc) (5). Vectors with correctly incorporated V(D)J gene transcripts were used for transient mammalian cell transfection (6) and the specificity was examined upon heterologous expression using a direct ELISA (7). OD A, optical density antigen; OD T, optical density transferrin (control). **(B)** Method 2: “Establishment of monoclonal EBV-LCLs”: Antigen-binding B cells were captured from B cells immortalized by Epstein-Barr Virus (EBV) using antigen-coupled magnetic beads (1 + 2). Based on theoretical frequency of antigen-binding B cells, cells were directly cloned by seeding 5 cells/well on top of irradiated PBMCs as feeder cells (3). After 4 weeks of culturing, the supernatants were checked for antibodies binding specifically to the antigen of interest (4). B cells with supernatant containing specific antibodies were seeded for an additional round of cloning (5) and the resulting supernatant was screened for specific antibodies (6) after additional 4 weeks of culturing. Monoclonality was checked by Sanger sequencing and the mAbs can be heterologously expressed as described for Method 1. LCLs, lymphoblastoid cell lines; OD A, optical density antigen; OD T, optical density transferrin (control).

This study provides a detailed description and comparison of two different strategies to generate human mAbs including single cell sequencing of antigen-binding B cells (Method 1) and the establishment of monoclonal EBV-immortalized B cell lines (Method 2). Both methods were used to generate human mAbs against peanut 2S albumins, major allergens in peanut allergy, and anti-Rho guanosine diphosphate dissociation inhibitor 2 (RhoGDI2, alternative and used ‘ARHGDIB’), a non-HLA target potentially involved in graft failure upon kidney transplantation (19). The generation of peanut 2S albumin-specific mAbs enables the evaluation of differences between specific antibody repertoires of peanut allergic and tolerant patients sensitized to these allergens. mAbs directed against ARHGDIB, on the other hand, enables the assessment of the role of ARHGDIB-specific mAbs in the pathogenesis of non-HLA related graft failure upon kidney transplantation, which can potentially lead to new therapeutic concepts in this context. Method 1 is especially suitable for a broad examination of antigen-binding mAbs due to a less selective process compared to Method 2. Method 2, however, is a powerful tool to select a small number of highly reactive mAbs potentially applicable in the development of treatment strategies. A schematic overview of both methods is shown in **Figure 1**.

## Materials

### Kits

EZ-Link™ Sulfo-NHS-Biotin, 21217, ThermoFisher Scientific, Waltham, Massachusetts, US
Pierce™ NHS-Activated Magnetic Beads, 88826, ThermoFisher Scientific, Waltham, Massachusetts, US
Transcriptor First Strand cDNA Synthesis Kit, 04379012001, Roche, Basel, Switzerland
Human B cell isolation kit II, 130-091-151, Miltenyi Biotec, Bergisch Gladbach, Germany
NucleoSpin gel and PCR clean up, REF 740609.250, Macherey Nagel, Düren, Germany
NucleoSpin Plasmid EasyPure, REF 740727.50, Macherey Nagel, Düren, Germany

### Enzymes

RNAse inhibitor, N8080119, ThermoFisher Scientific, Waltham, Massachusetts, US
RNAsin, N2515, Promega, Madison, Wisconsin, US
SuperScript™ III Reverse Transcriptase, 18080044, ThermoFisher Scientific, Waltham, Massachusetts, US
AmpliTaq Gold™ DNA polymerase, N8080249, ThermoFisher Scientific, Waltham, Massachusetts, US
Exonuclease I (*E. coli*), M0293L, New England BioLabs, Ipswich, Massachusetts, US
FASTAP Thermosensitive Alkaline Phosphatase, EF0651, ThermoFisher Scientific, Waltham, Massachusetts, US
Phusion high-fidelity DNA polymerase, M0530L, New England BioLabs, Ipswich, Massachusetts, US
AmpliTaq polymerase, N8080161, ThermoFisher Scientific, Waltham, Massachusetts, US
EcoRI restriction enzyme, R0101L, New England BioLabs, Ipswich, Massachusetts, US
NheI restriction enzyme, R0131L, New England BioLabs, Ipswich, Massachusetts, US
BsiWI restriction enzyme, R0553L, New England BioLabs, Ipswich, Massachusetts, US
AvrII restriction enzyme, R0174L, New England BioLabs, Ipswich, Massachusetts, US
T4 Polynucleotide kinase, M0201L, New England BioLabs, Ipswich, Massachusetts, US
T4 DNA ligase, M0202L, New England BioLabs, Ipswich, Massachusetts, US

### Antibodies

#### Antibodies for Single Cell Sorting

CD45-Pacific Orange, clone HI30, final dilution 1:40, MHCD4530, ThermoFisher Scientific, Waltham, Massachusetts, US
CD3-Pacific Blue, clone UCHT1, final dilution 1:160, 558117, BD Biosciences, East Rutherford, New Jersey, US
CD19-Fluorescein isothiocyanate, clone 4G7, final dilution 1:20, 345776, BD Biosciences, East Rutherford, New Jersey, US
CD14-R-phycoerythrin-cyanine dye Cy7, clone M5E2, final dilution 1: 800, 2109070, Sony Biotechnology, Inc., San Jose, California, US
CD16-R-phycoerythrin-cyanine dye Cy7, clone 3G8, final dilution 1:1000, 557744, BD Biosciences, East Rutherford, New Jersey, US
Streptavidin-Allophycocyanin, 17-4317-82, ThermoFisher Scientific, Waltham, Massachusetts, US
Streptavidin-R-phycoerythrin, 12-4317-87, ThermoFisher Scientific, Waltham, Massachusetts, US

#### Antibodies for ELISA

Goat anti-human IgE coupled with horse radish peroxidase (HRP), 5210-0158, KPL, Gaithersburg, Maryland, US:
Goat anti-human kappa antibody-HRP, 2060-05, SouthernBiotech, Homewood, Alabama, US
Goat anti-human lambda antibody-HRP, 2070-05, SouthernBiotech, Homewood, Alabama, US
Goat anti-human IgG FcY-HRP, 109-035-098, Jackson ImmunoResearch, Philadelphia, Pennsylvania, US
Goat anti-human IgG-PE antibody, 109-116-170, Jackson ImmunoResearch, Philadelphia, Pennsylvania, US
Donkey Anti-Goat IgG (H+L)-PE, 705-116-147, Jackson ImmunoResearch, Philadelphia, Pennsylvania, US

### Vectors

pFUSEss-CHIg-hE (hEF1-HTLV promotor, IL-2 signal sequence for secretion, multiple cloning site upstream the constant region and zeocin resistance gene for selection), pfusess-hche2, Invivogen, San Diego, California, US
pFUSEss-CHIg-hG1 (hEF1-HTLV promotor, IL-2 signal sequence for secretion, multiple cloning site upstream the constant region and zeocin resistance gene for selection), pfusess-hchg1, Invivogen, San Diego, California, US
pFUSEss-CLIg-hk (hEF1-HTLV promotor, IL-2 signal sequence for secretion, multiple cloning site upstream the constant region and blasticidin resistance gene for selection), pfuse2ss-hclk, Invivogen, San Diego, California, US
pFUSEss-CHIg-hl (hEF1-HTLV promotor, IL-2 signal sequence for secretion, multiple cloning site upstream the constant region and blasticidin resistance gene for selection), pfuse2ss-hcll, Invivogen, San Diego, California, US
pAdVAntage™ Vector, E1711, Promega, Madison, Wisconsin, US

### Cell Lines

Human embryonic kidney cells (HEK293-F (RRID : CVCL_6642), ThermoFisher Scientific, Waltham, Massachusetts, US
*E. coli* TOP10 cells, C404010, ThermoFisher Scientific, Waltham, Massachusetts, US
Irradiated (35 Gy) PBMCs, feeder cells for EBV-LCLs, freshly obtained from blood bank donors

### Medium

Fast-Media Zeo Agar (Lysogency Broth (LB) medium containing agar and zeocin), fas-zn-s, Invivogen, San Diego, California, US
Fast-Media Zeo Liquid (LB medium containing zeocin), fas-zn-s, Invivogen, San Diego, California, US
Fast-Media Blas Agar (LB medium containing agar and blasticidin), fas-bl-s, Invivogen, San Diego, California, US
Fast-Media Blas Liquid (LB medium containing blasticidin), fas-bl-s, Invivogen, San Diego, California, US
FreeStyle 293 expression medium, ThermoFisher Scientific, Waltham, Massachusetts, US
RPMI-1640, 11554526, ThermoFisher Scientific, Waltham, Massachusetts, US supplemented with 20% fetal calf serum and 1% Penicillin-Streptomycin

### Virus

Epstein-Barr virus was obtained from the supernatant of B95-8 cells, ACC 100, German Collection of Microorganisms and Cell Cultures GmbH, Braunschweig, Germany

### Antigens

Native peanut 2S albumins: isolated from roasted peanuts as described previously by Ehlers and colleagues (20)

ARHGDIB, heterologously expressed in HEK 293F cells, as described previously by Kamburova and colleagues (21)

## Methods

For the evaluation of both methods, we obtained blood from 14 blood bank donors who gave broad written informed consent at the research blood bank of the University Medical Center Utrecht (Mini Donor Dienst). Additionally, we included data from patients who were recruited for a study on peanut allergy and tolerance including 6 peanut-allergic, 6 peanut-tolerant patients and 5 non-atopic controls (obtained from the research blood bank) for single cell sorting of 2S albumin-binding B cells. Peanut allergic and tolerant patients gave written informed consent at the time point of inclusion. The corresponding study was approved by the Medical Ethical Committee at the University Medical Center Utrecht under the number 17-945 (20). An overview on which sample was used for which evaluation is shown **Table 1**.

**Table 1:** Patient samples used for each part of the protocol.

	Antigen	Patient samples
***Method 1: “Single cell sequencing of antigen-binding B cells”***		
*FACS set-up*	Peanut 2S albumins	4 blood bank donors†
*Amplification*		6 blood bank donors†
*Ab expression*		Study population
*FACS set-up*	ARHGDIB	3 blood bank donors‡
*Ab expression*		1 blood bank donor‡
***Method 2: “Establishment of monoclonal EBV-LCLs”***		
*Optimal seeding density*	Peanut 2S albumins	1 blood bank donor
*Cloning round 1*		2 blood bank donors§
*Cloning round 2*		2 blood bank donors§
*Cloning round 1*	ARHGDIB	2 blood bank donors¶
*Cloning round 2*		2 blood bank donors¶

^†^, ‡, §, ¶: indicating which samples were used for different steps

### Method 1: “Single Cell Sequencing of Antigen-Binding B Cells”

#### Antigen-Tetramer Formation

Isolated peanut 2S albumins (20) or heterologously expressed ARHGDIB (21) were treated with an excess of biotin in accordance with manufacturer’s instructions. The excess of biotin resulted in on average four biotin molecules per one molecule protein (EZ-Link™ Sulfo-NHS-Biotin). Antigen-tetramers were subsequently formed by separately adding streptavidin-R-phycoerythrin or streptavidin-Allophycocyanin to the biotinylated 2S albumin fraction or ARHGDIB in a molecular ratio of 1:1 (streptavidin: protein) (22, 23).

#### Single-Cell Sorting of Antigen Specific B Cells

Blood was drawn into heparin-coated tubes and freshly processed within 24 hours. Peripheral blood mononuclear cells (PBMCs) were obtained by density gradient centrifugation (Ficoll paque, SigmaAldrich) and they were finally resuspended in PBS containing 0.5% bovine serum albumin (BSA) and 2 mM EDTA. Subsequently, B cells were negatively enriched from the PBMCs fraction using antibody-coated magnetic beads (B cell isolation kit II). Enriched B cells were centrifuged (1000 x g, 20 °C, 5 min) and resuspended in PBS containing 0.5% BSA and 2 mM EDTA to obtain a concentration of 2*10^6 cells/40 µl. These B cells were stained for antigen specificity with 10 µl staining mix per 40 µl cell suspension using CD45-Pacific Orange (final dilution: 1:40), CD3-Pacific Blue (final dilution: 1:160), CD19-Fluorescein isothiocyanate (final dilution: 1:20), CD14-R-phycoerythrin-cyanine dye Cy7 (final dilution: 1:800), CD16-R-phycoerythrin-cyanine dye Cy7 (final dilution: 1:1000) and antigen-tetramers [75 pg^1^ 2S albumins, 1.1 ng^2^ ARHGDIB (24)]. Stained B cells were gated for CD45+, CD3-, CD19+, antigen tetramers (double positive) + and CD14/16- and single cell sorted into 96 wells fully-skirted PCR plates supplemented with 14 mM DTT and 11.2 U RNAse inhibitor in a total volume of 4 µl 0.5x PBS. Plates containing single-sorted B cells were immediately put on dry ice and stored at -80 °C until further processing. Before single cell sorting, the Aria II was calibrated to sort single cells into the middle of the respective well to minimize the probability that the sorted cell hits the wall.

**Figure 2:**
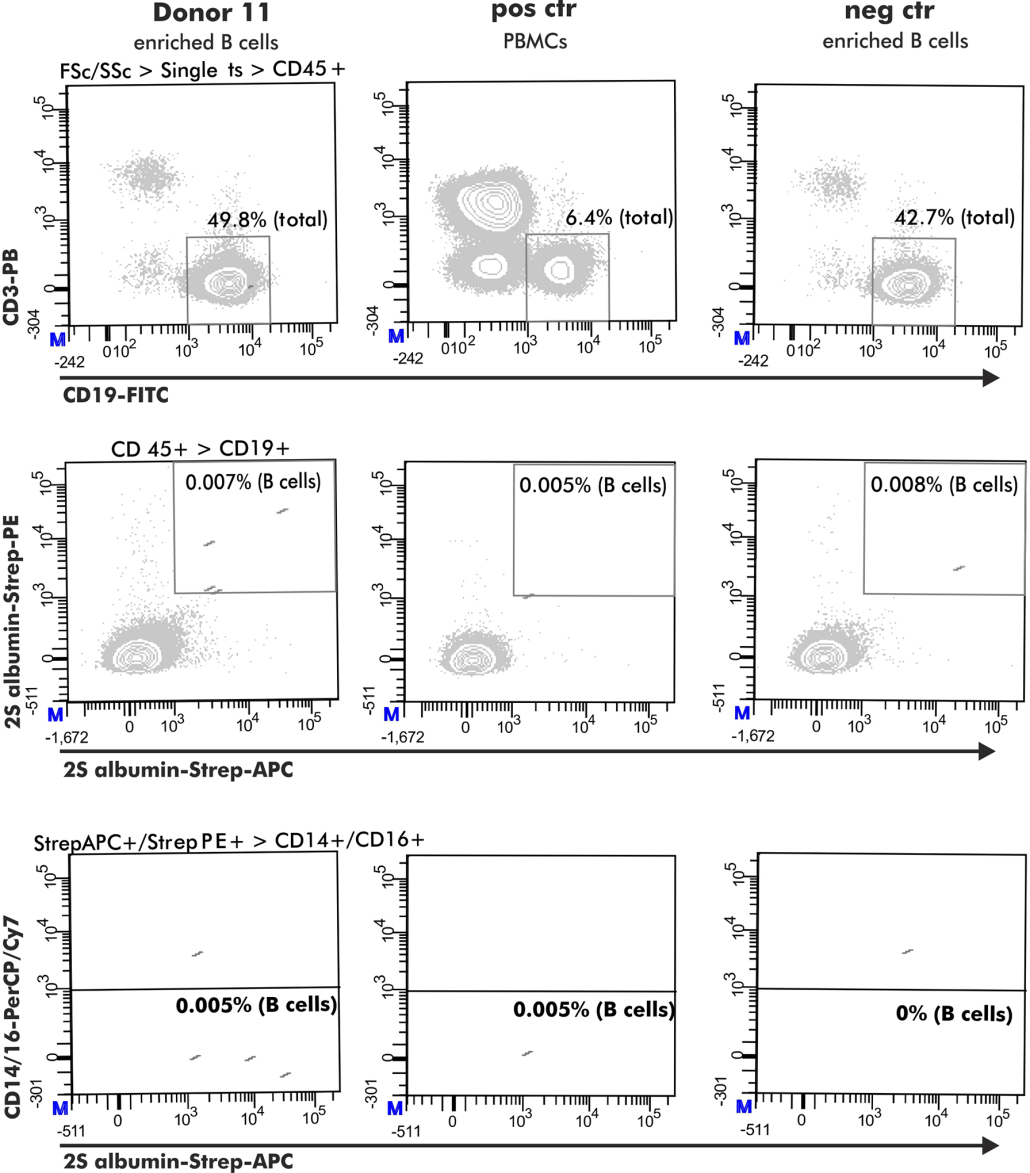
Flow cytometry gating strategy to obtain antigen-specific B cells. The selection of antigen-specific B cells (derived from a peanut-tolerant patient) was based on expression of CD45+ (CD45-PO), CD3- (CD3-PB), CD19+ (CD19-FITC) and double-positive binding to biotinylated antigen coupled with either streptavidin-PE or streptavidin-APC. This population must be negative for CD14 (CD14-PerCP/Cy7) and CD16 (CD16-PerCP/Cy7) expression.

For the selection of 2S albumin-binding B cells, a positive control comprising PBMCs from a healthy donor with detectable 2S albumin-binding B cells in previous experiments was included (healthy donor from the research blood bank but not belonging to the healthy donor pool used for analysis). B cells stained with biotin were used as negative control (Ctr 1). The gating was based on the respective negative control 1. An additional negative control (Ctr 2) was added to evaluate the optimal dilution of the used antigen-tetramers. This control consisted of a pre-incubation step with non-biotinylated antigen (75 pg 2S albumins, 1.1 ng ARHGDIB) followed by the normal staining protocol described above (**Figure 1A**, Step 1 and 2). The final gating strategy is shown in **Figure 2**.

#### Gene Amplification of B Cell Receptors

V(D)J gene transcripts amplification was performed as previous described with minor modifications (18, 22, 25). While keeping the frozen B cells on dry ice, they were supplemented with 1.4% NP-40, 3 U RNAse inhibitor and 7 µM hexamer primers reaching a total volume of 7.5 µl. For primer annealing, this mixture was subsequently incubated for one minute at 68 °C and cDNA transcription was performed in accordance with manufacturer’s instruction (SuperScript III). The resulting cDNA was subsequently used as template for the amplification of the heavy chain and its corresponding light chain gene transcript. Amplification was accomplished with 1.25 U hot-start DNA polymerase (AmpliTaq Gold), 2.5 mM MgCl_2_, 1 mM dNTP mix and 40 nM multiplex primers (**Table 2**) for 50 cycles. The annealing temperature was set to 62, 60 and 58 °C for heavy, κ and λ light chain, respectively (**Figure 1A**, Step 3).

**Table 2:** Primers used for single cell amplification, sequencing and cloning control; underlined: restriction sites; iltalic: additional nucleotide to ensure correct insertion.

Primer	Sequence
**Heavy chain primer forward**	
*Single cell RT-PCR*	
5’ L-Vh1	ACAGGTGCCCACTCCCAGGTGCAG
5’ L-Vh3	AAGGTGTCCAGTGTGARGTGCAG
5’ L-Vh4/6	CCCAGATGGGTCCTGTCCCAGGTGCAG
5’ L-Vh5	CAAGGAGTCTGTTCCGAGGTGCAG
*Introduction restriction sites*	
5’ EcoRI_VH1	ATATTGAATTC *G*CAGGTGCAGCTGGTGCAG
5’ EcoRI_VH1/5	ATATTGAATTC *G*GAGGTGCAGCTGGTGCAG
5’ EcoRI_VH1-18	ATATTGAATTC *G*CAGGTTCAGCTGGTGCAG
5’ EcoRI_VH1-24	ATATTGAATTC *G*CAGGTCCAGCTGGTACAG
5’ EcoRI_VH3	ATATTGAATTC *G*GAGGTGCAGCTGGTGGAG
5’ EcoRI_VH3-23	ATATTGAATTC *G*GAGGTGCAGCTGTTGGAG
5’ EcoRI_VH3-33	ATATTGAATTC *G*CAGGTGCAGCTGGTGGAG
5’ EcoRI_VH3-9	ATATTGAATTC *G*GAAGTGCAGCTGGTGGAG
5’ EcoRI_VH4	ATATTGAATTC *G*CAGGTGCAGCTGCAGGAG
5’ EcoRI_VH4-34	ATATTGAATTC *G*CAGGTGCAGCTACAGCAGTG
5’ EcoRI_VH4-39	ATATTGAATTC *G*CAGCTGCAGCTGCAGGAG
5’ EcoRI_VH6-1	ATATTGAATTC *G*CAGGTACAGCTGCAGCAG
*Sanger Sequencing*	
5’ Vh1-FR1_(1-2)	GGCCTCAGTGAAGGTCTCCTGCAAG
5’ Vh2-FR1_(2-5)	GTCTGGTCCTACGCTGGTGAAACCC
5’ Vh3-FR1_(3-7)	CTGGGGGGTCCCTGAGACTCTCCTG
5’ Vh4-FR1_(4-4)	CTTCGGAGACCCTGTCCCTCACCTG
5’ Vh5-FR1_(5-51)	CGGGGAGTCTCTGAAGATCTCCTGT
5’ Vh6-FR1_(6-1)	TCGCAGACCCTCTCACTCACCTGTG
*Colony screening*	
5’ Vh1-FR2_(1-2)	CTGGGTGCGACAGGCCCCTGGACAA
5’ Vh2-FR2_(2-5)	TGGATCCGTCAGCCCCCAGGGAAGG
5’ Vh3-FR2_(3-7)	GGTCCGCCAGGCTCCAGGGAA
5’ Vh4-FR2_(4-4)	TGGATCCGCCAGCCCCCAGGGAAGG
5’ Vh5-FR2_(5-51)	GGGTGCGCCAGATGCCCGGGAAAGG
5’ Vh6-FR2_(6-1)	TGGATCAGGCAGTCCCCATCGAGAG
5’ Vh7-FR2_(7)	TTGGGTGCGACAGGCCCCTGGACAA
**Heavy chain primer reverse**	
*Single cell RT-PCR*	
3’ CH1_IgA*	AGCCCTGGACCAGGCA
3’ CH1_IgE*,**	GAAGACGGATGGGCTCTGT
3’ CH1_IgG*,**	GGAAGGTGTGCACGCCGCTG
3’ CH1_IgM*	GGGAATTCTCACAGGAGACG
*Introduction restriction sites*	
3’ NheI_JH1/2/4/5	ATGCTAGCTGAGGAGACGGTGACCAG
3’ NheI_JH3	ATGCTAGCTGAAGAGACGGTGACCATTG
3’ NheI_JH6	ATGCTAGCTGAGGAGACGGTGACCGTG
**Kappa light chain primer forward**	
*Single cell RT-PCR*	
5’ L-Vκ1/2	ATGAGGSTCCCYGCTCAGCTGGTGG
5’ L-Vκ3	CTCTTCCTCCTGCTACTCTGGCTCCCAG
5’ L-Vκ4	ATTTCTCTGTTGCTCTGGATCTCTG
*Introduction restriction sites*	
5’ EcoRI_Vκ1-5	ATATTGAATTC *A*GACATCCAGATGACCCAGTC
5’ EcoRI_Vκ1-9	ATATTGAATTC *A*GACATCCAGTTGACCCAGTCT
5’ EcoRI_Vκ1D-43	ATATTGAATTC *A*GCCATCCGGATGACCCAGTC
5’ EcoRI_Vκ2-24	ATATTGAATTC *A*GATATTGTGATGACCCAGAC
5’ EcoRI_Vκ2-28	ATATTGAATTC *A*GATATTGTGATGACTCAGTC
5’ EcoRI_Vκ2-30	ATATTGAATTC *A*GATGTTGTGATGACTCAGTC
5’ EcoRI_Vκ3-11	ATATTGAATTC *A*GAAATTGTGTTGACACAGTC
5’ EcoRI_Vκ3-15	ATATTGAATTC *A*GAAATAGTGATGACGCAGTC
5’ EcoRI_Vκ3-20	ATATTGAATTC *A*GAAATTGTGTTGACGCAGTCT
5’ EcoRI_Vκ4-1	ATATTGAATTC *A*GACATCGTGATGACCCAGTC
*Colony screening*	
5’ Vk1f/6	TCAAGGTTCAGCGGCAGTGGATCTG
5’ Vk2f	GGCCTCCATCTCCTGCAGGTCTAGTC
5’ Vk3f	CCCAGGCTCCTCATCTATGATGCATCC
5’ Vk4_int	CAACTGCAAGTCCAGCCAGAGTGTTTT
5’ Vk5_int	CCTGCAAAGCCAGCCAAGACATTGAT
5’ Vk7_int	GACCGATTTCACCCTCACAATTAATCC
**Kappa light chain primer reverse**	
*Single cell RT-PCR*	
3’ Cκ 494*,**	GTGCTGTCCTTGCTGTCCTGCT
*Introduction restriction sites*	
3’ BsiWI_Jκ1/4	ATCGTACGTTTGATYTCCACCTTGGTC
3’ BsiWI_Jκ2	ATCGTACGTTTGATCTCCAGCTTGGTC
3’ BsiWI_Jκ3	ATCGTACGTTTGATATCCACTTTGGTC
3’ BsiWI_Jκ5	ATCGTACGTTTAATCTCCAGTCGTGTC
**Lambda light chain primer forward**	
*Single cell RT-PCR*	
5’ L-Vλ1	GGTCCTGGGCCCAGTCTGTGCTG
5’ L-Vλ2	GGTCCTGGGCCCAGTCTGCCCTG
5’ L-Vλ3	GCTCTGTGACCTCCTATGAGCTG
5’ L-Vλ4/5	GGTCTCTCTCSCAGCYGTTGCTG
5’ L-Vλ6	GTTCTTGGGCCAATTTTATGCTG
5’ L-Vλ7	GGTCCAATTCYCAGGCTGTGGTG
5’ L-Vλ8	GAGTGGATTCTCAGACTGTGGTG
*Introduction restriction sites*	
5’ EcoRI_Vλ1	ATATTGAATTC *G*CAGTCTGTGCTGACKCAG
5’ EcoRI_Vλ2	ATATTGAATTC *G*CAGTCTGCCCTGACTCAG
5’ EcoRI_Vλ3	ATATTGAATTC *G*TCCTATGAGCTGACWCAG
5’ EcoRI_Vλ4/5	ATATTGAATTC *G*CAGCYTGTGCTGACTCA
5’ EcoRI_Vλ6	ATATTGAATTC *G*AATTTTATGCTGACTCAG
5’ EcoRI_Vλ7/8	ATATTGAATTC *G*CAGRCTGTGGTGACYCAG
*Colony screening*	
5’ Vλ1/2_int	ATTCTCTGGCTCCAAGTCTGGC
5’ Vλ3_int	GGATCCCTGAGCGATTCTCTGG
**Lambda light chain primer reverse**	
*Single cell RT-PCR*	
3’ Cλ*,**	CACCAGTGTGGCCTTGTTGGCTTG
*Introduction restriction sites*	
3’ AvrII_Jλ1	ATTCCTAGGACGGTGACCTTGGT
3’ AvrII_Jλ2/3	ATTCCTAGGACGGTCAGCTTGGT
3’ AvrII_Jλ6	ATTCCTAGGACGGTCACCTTGGT
3’ AvrII_Jλ7-1	ATTCCTAGGACGGTCAGCTGGGT
3’ AvrII_Jλ7-2	ATTCCTAGGGCGGTCAGCTGGGT

*also used for Sanger Sequencing; **also used for colony screening.

**Note:** Primers aliquots (10 µM) should be stored in 5 mM Tris-HCl buffer, pH 8.0 instead of Milli-Q water to prevent degradation. Before using the primers for amplification, the primers are diluted 1:10 (1 µM) using Milli-Q water.

#### Sequence Analysis of B Cell Receptors

Amplified V(D)J gene transcripts were purified by adding 0.5 µl Exo RI (0.01 U) and 1 µl FAST-AP (1 U) and incubating this mixture for 30 min at 37 °C followed by 20 min at 80 °C. Purified heavy chain gene transcripts were Sanger sequenced by Macrogen service using 200 nM multiplex reverse or framework (FR)1 forward primers. Light chain gene transcripts were sequenced using 200 nM of the respective reverse primers (**Table 2**). To check the quality of the sequences, they were evaluated using Chromas Lite, version 2.6.5. Double peaks, potentially resulting from errors in the beginning of the amplification reaction, were aligned to their germline and corrected if plausible. Quality-checked gene sequences were saved as FASTA files and subsequently used for automatic germline alignment using the IgBLAST web interface (reference: IMGT database) (26). The resulting output was written into a SQLite database using R 3.6.3.

#### Cloning of V(D)J Gene Transcripts

To ensure the cloning of the entire V(D)J gene transcript without the introduction of additional amino acids, restriction sites were introduced with specific V gene forward and J gene reverse primers (**Table 2**). This introduction was achieved using 0.5 U Phusion high-fidelity DNA polymerase in presence of 1x reaction buffer, 2.5 mM MgCl_2_, 1 mM dNTP mix and 400 nM respective forward and reverse primers. The annealing temperature was set to 62, 60 and 58 °C for heavy, κ and λ light chain, respectively and the amplification reaction was performed for 30 cycles. Gene products, purified accordingly to manufacturer’s instructions (NucleoSpin gel and PCR clean up), were digested with 1 U of the respective restriction enzymes Eco RI and NhE I (heavy chain), BsWi (κ light chain) or Avr II (λ light chain). Before cloning the digested gene products into human IgH and IgL pFUSEss expression vectors, they were purified as already described and phosphorylated with 1 U T4-polynucleotide kinase for 60 min at 37 °C followed by 20 min at 65 °C. To prevent self-ligation, 1 µg of digested - 1 U respective restriction enzymes - and gel-purified parent vector was dephosphorylated with 4 U FAST-AP for 10 min at 37 °C followed by 10 min at 70 °C. Dephosphorylated vectors were mixed with digested gene products in a molecular ratio of 5:1 and incubated with 1 U T4 ligase for 1 hour at room temperature. The ligated vector was transformed into competent *E. coli* Top10 cells by allowing them to rest on ice for 30 min and subsequently, by incubating the mixture for 45 seconds at 42 °C (heat shock). Overnight grown colonies (LB Agar containing Zeocin or Blasticidin) were screened for incorporated V(D)J gene transcripts by PCR using 400 nM forward primers binding to the respective FR2 region and 400 nM reverse primers suitable for the constant part of the respective vector (**Table 2**) in presence of 2.5 U AmpliTaq polymerase, 2.5 mM MgCl_2_ and 1 mM dNTP mix. The annealing temperature was set to 63, 62 and 61 °C for heavy, κ and λ light chain, respectively and the amplification cycle was repeated for 30 times. Positive clones were grown overnight in 3 ml LB medium containing either zeocin or blasticidin. Purified vectors (NucleoSpin Plasmid EasyPure) were Sanger sequenced using the Macrogen service and the correctness was verified by aligning the vector sequence to the first sequence result (**Figure 1A**, Step 4 and 5).

#### Heterologous Expression of Monoclonal Antibodies

For heterologous expression of human mAbs, human embryonic kidney (HEK) 293F cells were cultured in FreeStyle 293 expression medium using 125 ml shaking culture flasks. Exponentially growing cells at a confluence of 80% and viability of 90% were transiently transfected with VH and VL expression vectors in a ratio of 2:3 (total 0.5 µg plasmid DNA per 1*10^6 cells) using 150 mm culture plates and 293fectin (2 µl/µg plasmid DNA, ThermoFisher Scientific). To ensure a sufficient intake, the expression vectors were supplemented with 0.5 µg pAdVAntage plasmid. The supernatant was harvested three days upon transfection and stored at -20 °C for further analyses (**Figure 1A**, Step 6).

### Method 2: “Establishment of Monoclonal EBV-LCLs”

#### Immortalization of Enriched B Cells by Epstein-Barr Virus

B cells, isolated from heparin blood as described for Method 1, were immortalized with EBV in presence of the TLR9 agonist CpG 2006 and the immunosuppressive Cyclosporine A to establish lymphoblastoid cell lines (LCLs) (15). In detail, 1 ml of EBV-containing supernatant, obtained from growing B-95.8 cells, was added to 5*10^6 pelleted B cells and incubated for 1 hour at 37 °C and 5% CO_2_. Upon washing with 1 ml PBS, infected B cells were resuspended in 3 ml RPMI-1640 supplemented with 20% fetal calf serum (FCS), 2.5 µg/ml CpG 2006, 1 µg/ml Cyclosporine A and 1% Penicillin-Streptomycin (Pen/Strep). The suspension was cultured at 37 °C and 5% CO_2_ until visible clusters were formed and the medium changed its color from red to yellow due to acidification (**Figure 1B**, Step 1).

#### Isolation of Antigen-Binding B Cells

Antigen-binding B cells were isolated from LCLs with an excess of antigen-coupled Pierce™ NHS-activated magnetic beads. The antigen was coupled to the beads in accordance with manufacturer’s instruction. For the isolation, LCLs (10*10^6 cells) were pelleted and cooled on ice for 60 min. The pellet was subsequently resuspended in 100 µl PBS supplemented with 0.5% BSA and 2 mM EDTA accompanied by 30 µl of antigen-coupled beads and cooled on ice for another 10 min. This suspension was applied on a magnetic column (MACS Cell Separation Columns, Miltenyi Biotec B.V.) and the separation was achieved in accordance with manufacturer’s instruction. The elution fraction was collected as antigen-binding B cells and was used to generate antigen-binding monoclonal LCLs (**Figure 1B**, Step 2).

#### Direct Cloning

The number of isolated antigen-binding LCLs was estimated based on the frequency of antigen-binding B cells determined by flow cytometry analysis (~ 0.01% of the B cells - Method 1). The elution fraction of the magnetic separation was mixed with 1*10^6/ml irradiated PBMCs (35 Gy) suspended in RPMI-1640 containing 20% FCS and 1% Pen/Strep to achieve a concentration of 25 antigen-binding LCLs/ml. 200 µl of this mixture was transferred to one well of a flat-bottom 96 wells plate (= 5 cells/well). The cells were allowed to grow for 4 weeks without re-feeding and the plate was tilted after the first week of culturing to keep the cells in close contact after the feeder cells died off. The plate was straightened again after an additional week of culturing to avoid too close contact between steadily proliferating cells (16) (**Figure 1B**, Step 3).

**Note:** It is crucial to tilt the 96 well plate after one week of culturing and straightening it again after 2 weeks of culturing.

#### Generating Monoclonal LCLs by Limiting Dilution Cloning

LCLs with supernatant containing antibodies specific to the antigen of interest were used for a second round of cloning to generate monoclonal LCLs. To this end, positive LCLs from the direct cloning step were counted and diluted to 50 cells/ml in RPMI-1640 supplemented with 20% FCS, 1% Pen/Strep and 1*10^6/ml irradiated PBMCs (35 Gy). This mixture was seeded in a volume of 200 µl/well in a flat-bottom 96 wells plate (10 cells/well) and incubated for 4 weeks as described above. For defining the optimal seeding density, a range from 0.3 to 10 cells/well was used.

Grown LCLs secreting antibodies specific for the antigen of interest were transferred to a 5 mL round bottom polystyrene test tube and cultured until a visible pellet was observed and the medium color changed from red to yellow. For further expansion, the LCLs were first transferred to a 25 cm^2^ culture flask and subsequently to a 75 cm^2^ culture flask. Expanded LCLs secreting specific mAbs were frozen at -80 °C and the supernatant containing mAbs was stored at -20 °C. Monoclonality was checked by Sanger Sequencing as described for Method 1 (**Figure 1B**, Step 4 to 6).

#### RNA Extraction

Total RNA was isolated accordingly to manufacturer’s instruction (RNA-Bee, BioConnect). Briefly, pelleted LCLs were homogenized in 1 ml RNA-Bee and the RNA was separated from the genomic DNA by adding 200 µl of chloroform and spinning for 15 min at 12.000 x g and 4 °C. The colorless phase was transferred to 500 µl ice-cold isopropanol and incubated for 10 min on ice to precipitate RNA. The precipitate was washed with 75% ethanol and the resulted pellet was resolved in RNAse free water for 15 min at 55 °C. Extracted RNA was either stored at -80 °C or used immediately.

#### cDNA Transcription and Gene Transcript Amplification

RNA was transcripted into cDNA in accordance with manufacturer’s instructions (Transcriptor First Strand cDNA Synthesis Kit). Briefly, 2 µl of random hexamer nucleotides (5 µM) were mixed with 8 µl of RNA template (1 µg) and the mixture was incubated for 3 min at 85 °C. Upon cooling down, 10 µl cDNA reaction mixture containing 1X RT-buffer, 5 mM MgCl_2_, 1 mM dNTP mix, 5U RNA inhibitor, 1.5 U avian myeloblastosis virus (AMV)-RT and 10 mM gelatin was added and incubated for 90 min at 42 °C. The reaction was stopped by inactivating the AMV-RT for 3 min at 85 °C. cDNA was either stored at -20 °C or immediately used for V(D)J gene transcript amplification as described for Method 1. Contrary to Method 1, the primers concentrations were adjusted to 400 nM instead of 40 nM.

### Examination of Specific Antigen-Binding

#### Specific Binding to Peanut 2S Albumins

Specificity of native and heterologously produced mAbs to peanut 2S albumins was tested using a direct ELISA. Briefly, plates were coated by applying either 0.3 µg/well Ara h 2 and 6 (27) or transferrin (negative control) overnight at room temperature. On the following day, the plate was blocked with PBS supplemented with 1% BSA and 0.1% Tween-20 (blocking buffer) for one hour at room temperature. Subsequently, supernatants were applied upon 1:2 dilution in blocking buffer (EBV LCLs) or in serial dilution (1 to 10 µg/ml) (heterologously expressed mAbs) for one hour at room temperature under continuous shaking. Bound antibodies from EBV-LCLs were detected with anti-human kappa (final dilution: 1:10.000) and anti-human lambda antibodies coupled with horse radish peroxidase (HRP) (final dilution: 1:5000) for one hour at room temperature under continuous shaking. Bound heterologously expressed mAbs, on the other hand, were detected by either goat anti-human IgE (final dilution: 1:5000) or goat anti-human IgG1 (final dilution: 1:2000) antibodies coupled with HRP under the same conditions. Visualization was provided by adding tetramethylbenzidine (TMB) for 15 minutes in the dark and the optical density (OD) was measured at 450 nm. Native Abs were considered for further cloning or analysis when the sample OD was at least two times higher than the OD of the negative control for round 1 and 2.5 times higher for round 2. Heterologously expressed mAbs were defined as specific if the sample OD was, upon subtraction of the negative control OD value, at least 1.5 times greater than the OD obtained with culture medium containing no mAbs. This evaluation was chosen, in comparison to the evaluation of native Abs, because the use of anti-IgE and anti-IgG-HRP as secondary antibodies resulted in higher background OD values when measuring binding to transferrin (coated mock antigen) compared with OD values to peanut 2S albumins as coated antigen. mAbs with OD values above 1 at a concentration of 10 µg/ml were considered as strong binders, mAbs with OD values between 0.07 and 1 at all concentration steps were considered as moderate binders and mAbs with increased OD values at the highest concentration of 10 µg/ml, but no detectable OD at the lowest concentration of 1 µg/ml were considered as weak binders.

#### Specific Binding to ARHGDIB

Antibody specificity to ARHGDIB was evaluated using ARHGDIB-coupled microspheres diluted in PBS supplemented with 0.1% BSA (wash buffer) (21). IgG-coupled microspheres served as positive control whilst empty and transferrin coupled microspheres served as negative controls. All incubation steps were performed in the dark, at room temperature and with continuous shaking.

For each antibody to be tested, 1500 microspheres consisting of 4 colors, each individually coated, were incubated with 50 µl undiluted HEK293 (Method 1) or EBV-LCLs supernatant (Method 2) containing the respective mAb and incubated overnight. Upon washing with a Bio-Plex Pro Wash station (Bio-Rad), bound mAbs were detected with either an 1-step (Method 1) or a 2-step procedure (Method 2). For the 1-step procedure, 50 µl of 1:50 diluted PE-conjugated goat-anti human IgG antibody was added and incubated for 30 min. For the 2-step procedure, 50 µl of goat anti-human kappa (final dilution: 1:100) and goat anti-human lambda antibody (final dilution: 1:32) was added and incubated for 30 min. For the second step, 50 µl of 1:100 diluted PE-conjugated donkey anti-goat IgG antibody was added and incubated for additional 30 min. For the readout of both procedures, 50 µl of washing buffer was added and the median fluorescence intensities (MFI) were measured on a Luminex 200 flow analyzer (Luminex Corp) (50 counts, 75 µl sample volume, 90s time out).

### Recovery Rate and Cost Calculation

The recovery rate of specific human mAbs was calculated for both methods as followed:

Method 1:

$$\begin{array}{c} Recovery\;rate=100\%*amlification\;efficiency*cloning\;efficiency*portion\;specific\;mAbs\\ =100\%*0.5*0.54*0.75\\ =20.3\% \end{array}$$

Method 2:

Recovery rate=n(wdcsells)∗efficiencydc∗n(mAbswdcsells)∗efficiencymcn(antigen−binding B cellstheo)∗100% = 200∗0.8∗2∗0.551000∗100%=17.6%

(dcs)wells= seeded wells for direct cloning based on theoretically antigen-binding B cells

*efficiency_dc_* = efficiency of direct cloning (approximate 2S albumins and ARHGDIB)

n(mAbswdcsells) = approximate number of mAbs obtained from one seeded well for direct cloning

*efficiency_mc_* = efficiency to obtain monoclonal cell lines upon 2nd round of cloning

Costs for the generation of one single human mAb was calculated by adding up the expenses and correcting it for the recovery rate of the respective strategy. Personal costs were included based on an average salary of a research technician and the approximate working hours needed to produce one single mAb.

## Results

### Method 1: “Single Cell Sequencing”

#### Double Tetramer-Staining Reduced the Selection of Non-specific CD19+ B Cells

**Figure 3:**
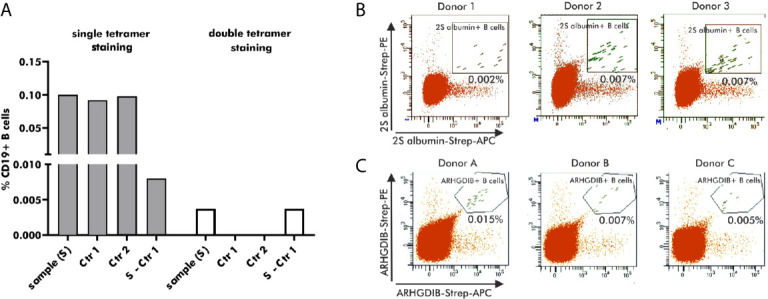
Selection of antigen-binding B cells using antigen-tetramers. **(A)** Percentage of 2S albumin-binding B cells (blood bank donors) in relation to the total CD19+ B cell fraction using single or double-tetramer staining. Control 1 (Ctr 1) represents the staining with only biotin and Control 2 (Ctr 2) is executed by pre-blocking with unlabeled 2S albumin prior normal staining procedure. The final percentage is calculated by subtracting Ctr 2 from the sample. **(B, C)** FACS plots representing antigen-binding B cells in relation to the total B cell population of 6 independent blood bank donors. 2S albumin-binding B cells are shown in **(B)** and ARHGDIB-binding B cells in **(C)**.

Antigen-binding B cells for subsequent single cell V(D)J gene transcript amplification were detected by flow cytometry using antigen-tetramers formed with fluorophore-labeled streptavidin. For the development of an optimal staining protocol, enriched B cells, derived from the same blood bank donor, were stained with 2S albumin-tetramers accompanied by either a single (APC) or two distinct fluorophores (APC and PE). As shown in **Figure 3A**, B cells single-positive for 2S albumin-tetramer binding accounted for 0.1% of the total CD19+ B cell fraction. Both controls - staining with biotin without the antigen (Ctr 1) and blocking with unlabeled antigen (Ctr 2) - showed, however, a comparable percentage of 2S albumin-binding B cells (Ctr 1: 0.092%; Ctr 2: 0.097%). The subtraction of Ctr 1 resulted in a final percentage of 0.008% 2S albumin-binding B cells. In comparison, the fraction of double-positive 2S albumin-binding B cells was already reduced to a percentage of 0.004% without any background staining, indicating the potential of double antigen-tetramer staining for identifying bona fide antigen-binding B cells. For validation purposes, double-tetramer staining was used for the detection of 2S albumin (**Figure 3B**) or ARHGDIB-binding B cells (**Figure 3C**) in 3 independent blood bank donors, respectively (n=6, **Table 1**). 2S albumin-binding B cells ranged from 0.002 to 0.007% whilst ARHGDIB-binding B cells ranged from 0.005 to 0.015%, indicating a good reproducibility of the developed staining protocol.

#### V(D)J Gene Transcript Amplification Efficiency Is Donor-Dependent

Amplification efficiency upon single cell sorting of 2S albumin-binding B cells was evaluated in 6 independent blood bank donors. Amplification efficiency of the heavy chain VDJ gene transcript from 24 to 50 individual B cells ranged from 29 to 63% whilst the percentage of successfully amplified V(D)J gene transcripts from the heavy and corresponding light chain was reduced and ranged from 17 to 50%. Although a low amplification efficiency was shown for two donors, most of the donors (4/6) showed sufficient amplification efficiencies of approximately 50%^3^. Cloning of successfully amplified heavy and corresponding light chain gene transcripts from peanut allergic, peanut tolerant and non-atopic controls resulted into an overall cloning efficiency of 54% which may be increased by ordering the not successfully cloned gene sequences commercially. Individual cloning efficiencies were estimated to 76% for the heavy chain gene transcripts and 71% for the light chain gene transcripts (78% kappa and 31% lambda). In the present study, 4 gene sequences, whose cloning originally failed, were commercially obtained and the cloning of those sequences were subsequently successful. Hence, the overall cloning efficiency might be increased to 76% when obtaining gene sequences partly commercially.

#### Antibodies Derived From Double-Positive Tetramer-Binding B Cells Are Mostly Specific

The specificity of human mAbs, generated by single cell sequencing, can only be examined upon heterologous expression in the end of the workflow. Overall, 33 heterologously expressed mAbs from 10 different donors carried the variable region of B cells selected for their putative binding to peanut 2S albumins and 1 mAb carried the variable region of one B cell selected for putative binding to ARHGDIB. These mAbs were randomly expressed as IgE or IgG1 mAbs as this enabled the assessment of their potency to induce degranulation (IgE) and their ability to block serum IgE binding (IgG1) in the study of Ehlers and colleagues (20). Their concentrations varied between 0.1 and 10 µg/ml.

**Figure 4:**
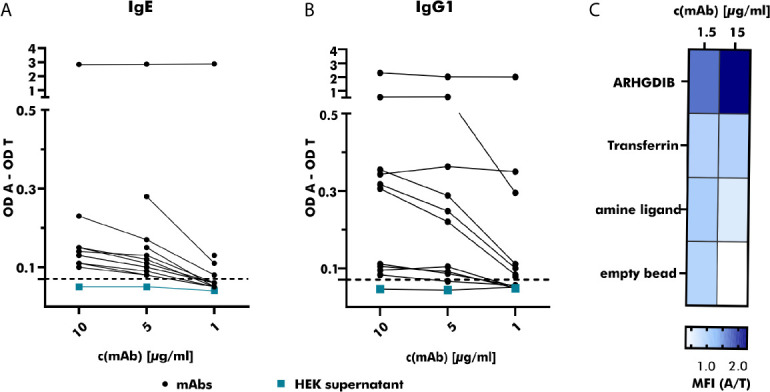
Specific binding of produced monoclonal antibodies towards the respective antigen. **(A)** The binding of serial-diluted (1 to 10 µg/ml) human monoclonal antibodies (mAbs) (expressed as IgE; derived from peanut-allergic and peanut-tolerant patients) towards peanut 2S albumins was measured using anti-human IgE-horse radish peroxidase (HRP) as detection antibody. Measured optical density (OD) values were corrected by subtracting the OD value measured for the control antigen (transferrin) and compared to the OD value obtained with HEK supernatant not containing any antibodies (turquoise). The threshold was set to an OD value 1.5 times greater than the OD value obtained with HEK supernatant containing no mAb. OD A, optical density antigen; OD T, optical density transferrin (control). **(B)** The binding of serial-diluted (1 to 10 µg/ml) human mAbs (expressed as IgG1, derived from peanut-allergic and peanut-tolerant patients) towards peanut 2S albumins was measured using anti-human IgG-HRP as detection antibody. The measured OD value was corrected as described for **(A)**. **(C)** The specificity of the mAb derived from an ARHGDIB-binding B cell, derived from a blood bank donor, was examined using ARHGDIB-coupled microspheres. The median fluorescence intensity (MFI) towards ARHGDIB was evaluated in relation to the MFI towards transferrin as control antigen. A, antigen (ARHGDIB); T, transferrin.

Binding to 2S albumins was observed in 75% (25/33) of all heterologously expressed mAbs. While mAbs with the ability to specifically bind to peanut 2S albumins originated from 11 IgM+, 9 IgG+ and 5 IgA+ B cells, all (n=8) mAbs without specific binding to peanut 2S albumins originated from IgM+ B cells. Based on their binding abilities, examined by comparing their achieved OD values at different concentrations, they were roughly categorized into weak (n=13), moderate (n=8) and strong (n=2) binders (**Figures 4A, B**). This variability in target binding indicates that our selection strategy was not restricted to only strongly binding B cells and implicates no selection bias regarding distinct affinities. Our staining protocol can also be adjusted to different antigen targets as the mAb generated from an ARHGDIB-binding B cell (IgA+) showed strong binding to ARHGDIB-coupled microspheres with an ARHGDIB/transferrin ratio greater than 2 (**Figure 4C**).

### Method 2: “Establishment of Monoclonal EBV-LCLs”

#### LCLs Isolated With Antigen-Coupled Beads Are Mostly Antigen-Specific

**Figure 5:**
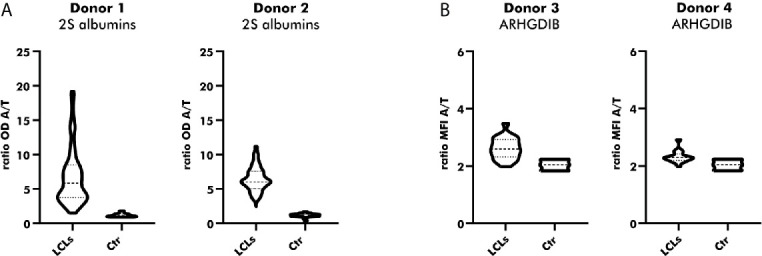
Direct cloning of peanut 2S albumin and ARHGDIB-binding EBV-LCLs from blood bank donors. **(A)** Antibodies secreted by directly cloned EBV-LCLs were screened for their binding to peanut 2S albumins using a direct ELISA. EBV-LCLs secreting antibodies with an OD ratio (2S albumin/transferrin) ≥ 2 were selected for an additional round of cloning. The data are presented as violin blot with the median and the inter-quartile range. Control (Ctr): irradiated PBMCs without EBV-LCLs. LCLs, lymphoblastoid cell lines; OD, optical density; A, antigen; T, transferrin (mock antigen). **(B)** Antibodies secreted by directly cloned EBV-LCLs were screened for their binding to ARHGDIB using antigen-coupled microspheres. EBV-LCLs secreting antibodies with a MFI ratio (ARHGDIB/transferrin) ≥ 2 were selected for an additional round of cloning. The data are presented as violin blot with the median and the inter-quartile range. Control (Ctr): irradiated PBMCs without EBV-LCLs. LCLs, lymphoblastoid cell lines; MFI, median fluorescent intensity; A, antigen; T, transferrin (mock antigen).

Native Abs can be produced by *in-vitro* culturing of primary B cells secreting polyclonal Abs upon activation. Since *in-vitro* culturing of primary B cells is challenging (28), especially in the absence of a particular antigen, we chose for the establishment of LCLs by EBV immortalization. Immortalized LCLs were selected for their binding to either peanut 2S albumins or ARHGDIB. As shown in **Figure 5A**, almost all polyclonal EBV-LCLs (98.8%) obtained from the first round of cloning secreted antibodies specifically binding to 2S albumins (2S albumin/transferrin ratio ≥ 2) compared to the supernatant of irradiated PBMCs (Ctr). This large number of positive EBV-LCLs (100%) was confirmed by selecting for 2S albumin-binding EBV-LCLs using a second blood bank donor. Although the number of positive clones was reduced for ARHGDIB-binding B cells (Donor 1: 67%, Donor 2: 56%), a representative number of EBV-LCLs was selected for a second round of cloning to achieve monoclonality (**Figure 5B**).

#### Optimal Seeding Density to Achieve Monoclonal LCL Clones

**Figure 6:**
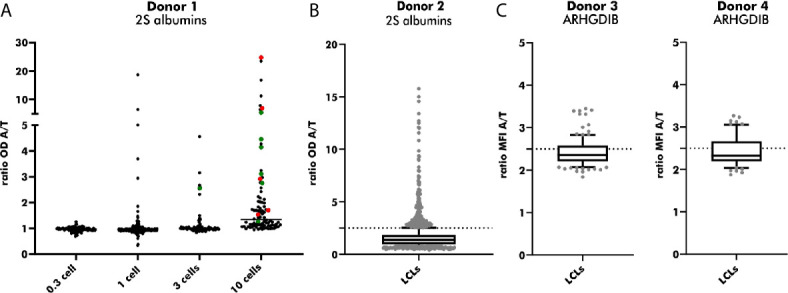
Subcloning of 2S albumin and ARHGDIB-binding EBV-LCLs from blood bank donors. **(A)** Determination of the optimal seeding density for the second round of EBV-LCLs cloning. EBV-LCLs with specific binding to peanut 2S albumins (ratio 2S albumins/transferrin ≥ 2) were considered for expansion. The V(D)J gene transcript of these EBV-LCLs were Sanger sequenced to check for monoclonality. Green: monoclonal EBV-LCLs, red: polyclonal EBV-LCLs, black: not sequenced. LCLs, lymphoblastoid cell lines; OD, optical density; A, antigen; T, transferrin (mock antigen). **(B)** Antibodies secreted by subcloned EB V-LCLs were screened for their binding to peanut 2S albumins. EBV-LCLs with a ratio (2S albumin/transferrin) ≥ 2.5 were considered for expansion. The data are presented as box blot with the median and the inter-quartile range. The lowest and highest 10% are presented as grey, filled dots. LCLs, lymphoblastoid cell lines; OD, optical density; A, antigen; T, transferrin (mock antigen); dash line: set threshold to ratio ≥ 2.5. **(C)** Antibodies secreted by subcloned EBV-LCLs were screened for their binding to ARHGDIB. EBV-LCLs with a ratio (ARHGDIB/transferrin) ≥ 2.5 were considered for expansion. The lowest and highest 10% are presented as grey, filled dots. LCLs, lymphoblastoid cell lines; MFI, median fluorescent intensity; A, antigen; T, transferrin (mock antigen); dash line: set threshold to ratio ≥ 2.5.

LCLs positive for antigen-binding were used for an additional round of cloning to achieve LCLs secreting monoclonal instead of polyclonal Abs. EBV-LCLs from the first blood bank donor were used to determine the optimal seeding density. Even though a seeding density of 0.3 cells/well results theoretically in the highest probability of obtaining monoclonal LCLs, only a small number of wells contained LCLs showing proliferation. By increasing the seeding density, the number of LCLs secreting Abs binding to 2S albumins rose in accordance with the number of LCLs seeded per well. Despite a high seeding density of 10 cells/well, 55% of sequenced EBV-LCLs (6/11) showed monoclonality, leading to a compromise between a high number of proliferating EBV-LCLs and a reasonable rate of achieved monoclonal EBV-LCLs (**Figure 6A**). Seeding 2S albumin-binding EBV-LCLs from the second blood bank donor at a density of 10 cells/well resulted in an approximate recovery of 2 EBV-LCLs with specific binding to 2S albumins per seeded plate (**Figure 6B**) when setting the threshold to a ratio (OD antigen/OD transferrin) of 2.5. This increased threshold was set to enlarge the probability to select truly specific LCLs combined with a certain amount of secreted antibodies. A comparable recovery was achieved for ARHGDIB-binding EBV-LCLs from another two independent blood bank donors (**Figure 6C**).

#### Heterologously Expressed mAbs Showed Comparable Binding to 2S Albumins

**Figure 7:**
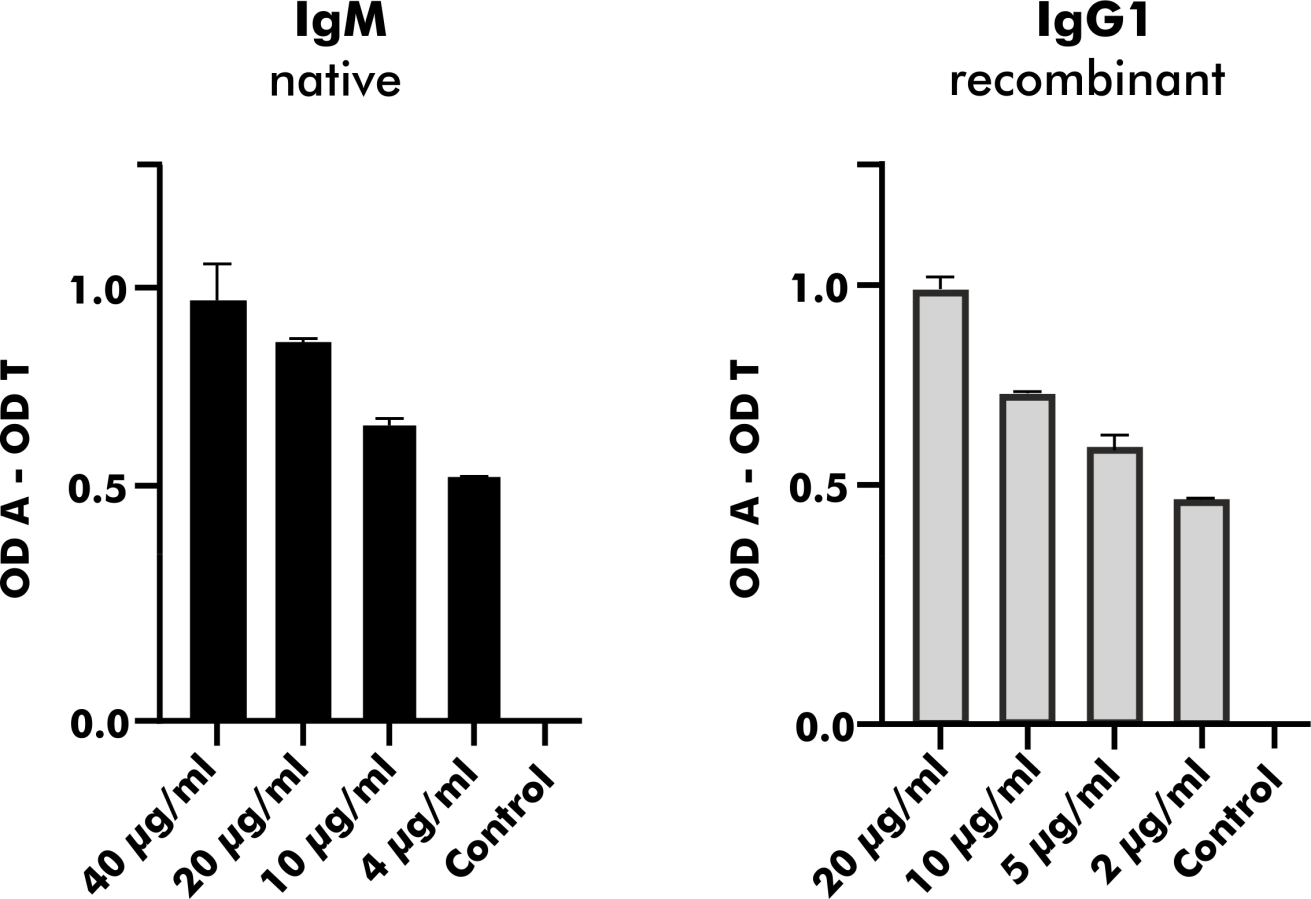
Heterologous expression of a human mAb with specific binding to peanut 2S albumins derived from a blood bank donor. A mAb carrying the same variable region as native antibodies obtained from a monoclonal EBV-LCL was heterologously expressed with a vector containing the IgG1 backbone. Its binding ability expressed as OD values (corrected for binding to transferrin) was compared to the binding ability of its native counterpart (IgM) using anti-kappa-HRP as secondary antibody. The data are presented as the mean with the standard deviation of two replicates. OD, optical density; A, antigen; T, transferrin (mock antigen).

To validate heterologous expression of mAbs secreted by established EBV-LCLs, one pair of heavy and corresponding light chain variable regions was selected from the pool of 2S albumin-specific monoclonal EBV-LCLs. Upon heterologous expression with a vector containing the IgG1 backbone, native and heterologously expressed counterparts were applied on a direct ELISA in a serial dilution as shown in **Figure 7**. The measured OD values were comparable between the native IgM mAb and the heterologously expressed IgG1 mAb (light chain detection), indicating that mAbs from subcloned EBV-LCLs can also be heterologously expressed with comparable binding abilities.

#### Single Cell Sequencing Allows Higher Throughput

Both methods described within this chapter are characterized by several up- and downsides influencing the choice which method to use. A comparison regarding costs, expenditure of time, recovery, throughput and complexity is shown in **Table 3**. Without considering any further characterization, the generation of one single human mAb can add up to 750€. The establishment of monoclonal EBV-LCLs, however, is generally less expensive (400 - 500€) compared with the generation by single sequencing (650 - 750€), resulting from expensive reagents required for single cell amplification. On the other hand, the workflow of single cell sequencing shortens the required time from 12 weeks including long culturing periods to only 3 weeks. Even though both methods showed comparable recovery rates in our experiments, the establishment of monoclonal EBV-LCLs reaches easily a capacity threshold limiting the overall throughput and leading to random selection of clones to proceed with. Hence, the establishment of monoclonal EBV-LCLs is a suitable tool to generate a limited number of human mAbs, especially due to the ability to screen for binding, functionality and neutralization capacity throughout the workflow. Single cell sequencing is, however, a more suitable tool for a broader examination of antigen-specific B cells, since a much higher throughput can be achieved. Unfortunately, specificity and functionality can only be examined in the end of the workflow. Both methods are laborious and highly complex.

**Table 3:** Comparison of Method 1 and 2 to generate human mAbs.

	Method 1: “Single cell”	Method 2: “EBV-LCLs”
*Costs	*~650 - 750€	*~450€
*Time*	3 weeks	12 weeks
*Antigen adaptation*	sorting strategy: approx. 3 weeks	No special adaptation needed (only antigen coupling to beads – 1 day)
*Recovery*	20.3 - 29%**	17.6%***
*Throughput*	+++	+
*Complexity*	+++	+++

*costs per one single human mAb. **Cloning efficiency might be increased from 54% to 76% by purchase gene sequences commercially. ***The number of subcloned B cells is reduced by reaching a certain capacity threshold. This has been reflected with the limited throughput. +: low, ++: moderate, +++: high.

## Discussion

Studying human-derived mAbs offers the possibility to link genetic information to functional features, making them indispensable in modern molecular biology research. In this study, we directly compared two distinct strategies to generate (highly) specific mAbs from peripheral blood of human donors, i.e.: single cell sequencing and the establishment of monoclonal EBV-LCLs. While single cell sequencing is a suitable tool to generate a large panel of mAbs with distinct binding features, the establishment of monoclonal EBV-LCLs provides the possibility to screen for specificity and functionality throughout the workflow. Both strategies, initially set-up for mAbs specifically directed against peanut 2S albumins, were easily adaptable to other antigen targets as shown for ARHGDIB.

Corresponding V(D)J gene transcripts were successfully amplified from up to 50% of all single cell sorted 2S albumin-binding B cells. The overall recovery of 2S albumin-specific human mAbs, however, was reduced to around 20% by taking the cloning efficiency (54%) and proportion of mAbs with proven specificity (75%) into consideration. The cloning efficiency may be increased by ordering gene sequences without cloning success commercially. Overall, our amplification efficiency (~50%) corresponds to the work of Tiller and colleagues who described an amplification efficiency of up to 60% (18). Moreover, a comparable overall recovery of 27% has been shown for single cell sequencing of antigen-specific B cells from guinea pigs (29). Increased amplification efficiencies of 90 to even 100% have been described for performing a comparable amplification protocol in triplicates (30). However, such an approach will simultaneously increase the probability of amplification errors, potentially hampering gene analysis and antibody cloning.

Regarding the establishment of antigen-specific monoclonal EBV-LCLs, the overall recovery was estimated to around 18%. This is in accordance with cloning efficiencies of around 15% observed for limiting dilution approaches upon EBV immortalization (16). However, we lack the information what proportion of antigen-specific B cells were initially immortalized by EBV. To overcome this limitation, Fraussen and colleagues described in their protocol the immortalization of 50 cells/well upon sorting the desired B cell subpopulation (31). However, we were not able to immortalize such small numbers of B cells successfully in our laboratory.

Both strategies are characterized by their own strengths and limitations. Single cell sequencing, on the one hand, enables the execution in a moderate-throughput manner and the selection of antigen-specific B cells is not restricted to certain subpopulations as EBV immortalization is restricted to CpG-activated memory B cells (15). These advantages make the single cell sequencing platform a suitable tool for a broad examination of antigen-binding B cells and their corresponding mAbs. The establishment of monoclonal EBV-LCLs, on the other hand, provides continuous screening for specific binding and functionality throughout the workflow, resulting in an easy selection of mAbs with high affinity towards their targets and making this strategy a powerful tool in therapeutic research. Moreover, this strategy enables the comparison of heterologously expressed mAbs with their natural counterparts, allowing the identification of potential structure alterations by post-translational modifications during heterologous expression (32, 33). An additional advantage of generating human mAbs by establishing monoclonal EBV-LCLs is their limited need for expensive reagents.

In conclusion, both strategies - single cell sequencing and establishment of monoclonal EBV-LCLs - are able to generate (highly) antigen-specific human mAbs and they are easily adaptable to other target antigens. The recommended method to choose is dependent on the research question to explore as both strategies have their own strengths and limitations.

## Data Availability Statement

All data are presented in this article. Genetic information about the generated antibodies can be found under https://www.ncbi.nlm.nih.gov/genbank/, MW271045 - MW271525.

## Ethics Statement

The studies involving human participants were reviewed and approved by Medical Ethical Committee of the University Medical Center Utrecht. The patients/participants provided their written informed consent to participate in this study.

## Author Contributions

AE, CHJ, and HO: experimental design. AE, CHJ, TK-H, and MK: experimental performance. AE, CHJ, TK-H, and MK: data collection and analyses. AE: drafting the manuscript. AE, CHJ, TK-H, MK, AK, and HO: contribution to data interpretation. CHJ, TK-H, MK, AK, and HO: critical revision of the manuscript. All authors contributed to the article and approved the submitted version.

## Funding

For financial support, we want to acknowledge EUROIMMUN AG, Lübeck, Germany.

## Conflict of Interest

The research position of AE was partially funded by EUROIMMUN AG, Lübeck, Germany.

The remaining authors declare that the research was conducted in the absence of any commercial or financial relationships that could be construed as a potential conflict of interest.

## Footnotes

1 ≙ 30*10^6 antigen-tetramers sufficient for approximately 150-300 resting B cells.

2 ≙ 112*10^6 antigen-tetramers sufficient for approximately 500-1000 resting B cells.

3 Note: We observed higher amplification efficiencies when primer aliquots were stored in 50 mM Tris-HCl buffer supplemented with 2 mM EDTA instead of RNAse free water.
